# A blinking periorbital prosthesis using surface electromyographic signals of the orbicularis oculi muscle

**DOI:** 10.1007/s00238-015-1111-4

**Published:** 2015-06-03

**Authors:** Tadashi Akamatsu, Norimichi Kawashima, Takeshi Tsunekuni, Kotaro Imagawa, Muneo Miyasaka

**Affiliations:** Department of Plastic and Reconstructive Surgery, School of Medicine, Tokai University, 143 Shimokasuya, Isehara, Kanagawa, Japan; International Institute for Science and Education, International Pacific University, 1-1-7 Sakuragicho, Yokohama, Kanagawa, Japan; Ahead Laboratories Inc., 1-2-14 Shinkawa, Chuo, Tokyo, Japan

**Keywords:** Prosthesis, Blinking, Myoelectric potential, Surface EMG

## Abstract

**Background:**

Recent advances in human–machine interface technology have enabled the development of multifunctional, primarily orthopedic myoelectric prostheses. We developed a noninvasive blinking periorbital prosthesis that can synchronize with blinking of the intact eyelid by using surface electromyographic signals of the orbicularis oculi muscle.

**Methods:**

Myoelectric potentials of the orbicularis oculi muscle while blinking were measured with surface electrodes on the eyelid in four healthy adults. Possible cross talk introduced via the electrodes was also measured and assessed to determine whether cross talk would affect surface electromyographic measurements while blinking.

**Results:**

The amplitude of the surface myoelectric potential of the orbicularis oculi muscle was sufficiently high for the practical use of blinking prostheses. Our blinking model was successfully synchronized with blinks of the subjects’ eyelids under experimental conditions without cross talk between the orbicularis oculi muscle and other muscles.

**Conclusions:**

Although our study revealed several problems, the use of surface electromyographic signals could be a promising and useful technique for synchronizing blinking of the prosthetic eyelid with blinking of the intact eyelid.

Level of Evidence: Level V, therapeutic study.

**Electronic supplementary material:**

The online version of this article (doi:10.1007/s00238-015-1111-4) contains supplementary material, which is available to authorized users.

## Introduction

Recent advances in human–machine interface technology have enabled the development of multifunctional myoelectric prostheses as reported mainly in the orthopedic field [1–4]. In the cranio-maxillofacial surgery field, reconstructive treatment with silicon facial prostheses is still indicated in some patients with large periorbital tissue defects [5–7]. Several researchers have developed orbital prostheses with eyelid movements to improve the unnatural appearance of the prosthesis [8, 9].

We have been developing a blinking periorbital prosthesis that can be synchronized with blinking of the intact eyelid using electromyographic (EMG) signals detected noninvasively from the skin surface above the orbicularis oculi muscle. Various human–machine interface technologies exist to detect the patient’s movement intention. Such technologies typically use EMG signals, electroencephalographic (EEG) signals through scalp or intracranial electrodes [10, 11], or a combination of these systems [12]. Among these signals, surface EMG signals are used in most prosthetic limbs that are already in practical use in the orthopedic field [4]. Therefore, we consider surface EMG signals to be the most promising approach to develop blinking maxillofacial prostheses that can be used by patients in real-life conditions.

In the present study, we developed a blinking model that can synchronize with blinking of the intact eyelid using surface EMG signals of the orbicularis oculi muscle. In addition, we determined whether surface EMG detection at blinking would be affected by cross talk of other facial muscles due to facial or head movements. We did not study the application of our model to a wearable periorbital prosthesis.

## Materials and methods

### EMG measurement of the orbicularis oculi muscle and selection of the recording electrode position

**Fig. 1:**
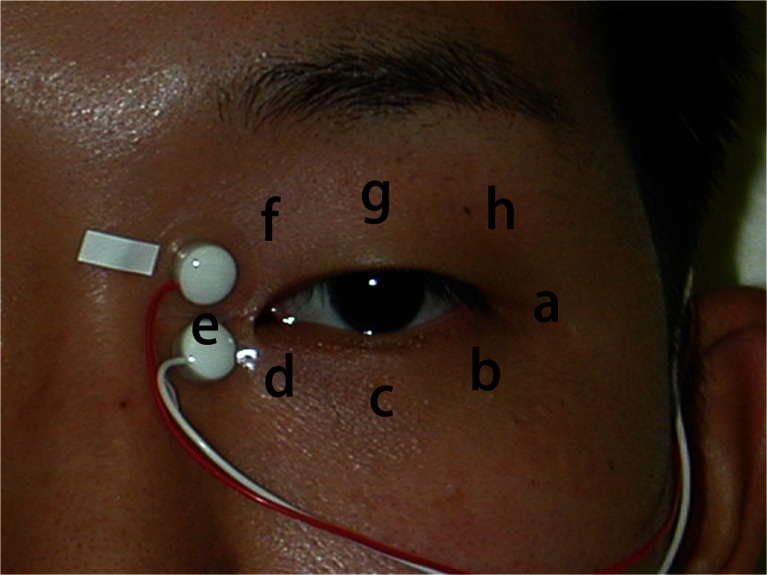
Surface EMG signals of the orbicularis oculi muscle were measured at the following eight electrode positions: lateral canthus (*position a*), lateral lower eyelid (*position b*), central lower eyelid (*position c*), medial lower eyelid (*position d*), medial canthus (*position e*), medial upper eyelid (*position f*), central upper eyelid (*position g*), and lateral upper eyelid (*position h*)

To select a recording electrode position for detection of EMG signals while blinking, surface EMG was measured at eight electrode positions above the orbicularis oculi muscle (Fig. 1). The study subjects were four healthy adults (two men and two women aged 21–24 years).

A pair of biological electrodes (NT-211U and NT-214U, Nihon Kohden Corporation, Shinjuku, Tokyo, Japan) was placed at one of the eight positions on the skin, parallel to the muscle fiber direction of the orbicularis oculi muscle. The interval between the electrodes was 8 mm. The detected EMG signals were amplified 1000-fold with an amplifier (BA1104CM, TEAC Corporation, Tama, Tokyo, Japan). The amplified signals were recorded, analyzed, and displayed with a EMG waveform analysis software (KM104, Ozawa Medical Instruments Co., Ltd., Kurashiki, Okayama, Japan) on a laptop computer running a Windows operating system (Microsoft Corporation, Redmond, WA, USA). All four subjects provided their informed consent prior to their inclusion in the study.

### Assessment of EMG cross talk

The following four possible sources of cross talk were assessed by measuring EMG signals of the neighboring muscles simultaneously with those of the orbicularis oculi muscle while blinking: (1) cross talk with the masticatory muscles due to strong clenching, (2) cross talk with the extraocular muscles and frontalis muscles due to ocular movements, (3) cross talk due to a vertical or horizontal headshake, and (4) cross talk due to speaking.

### Operation verification of the prosthesis model

**Fig. 2:**
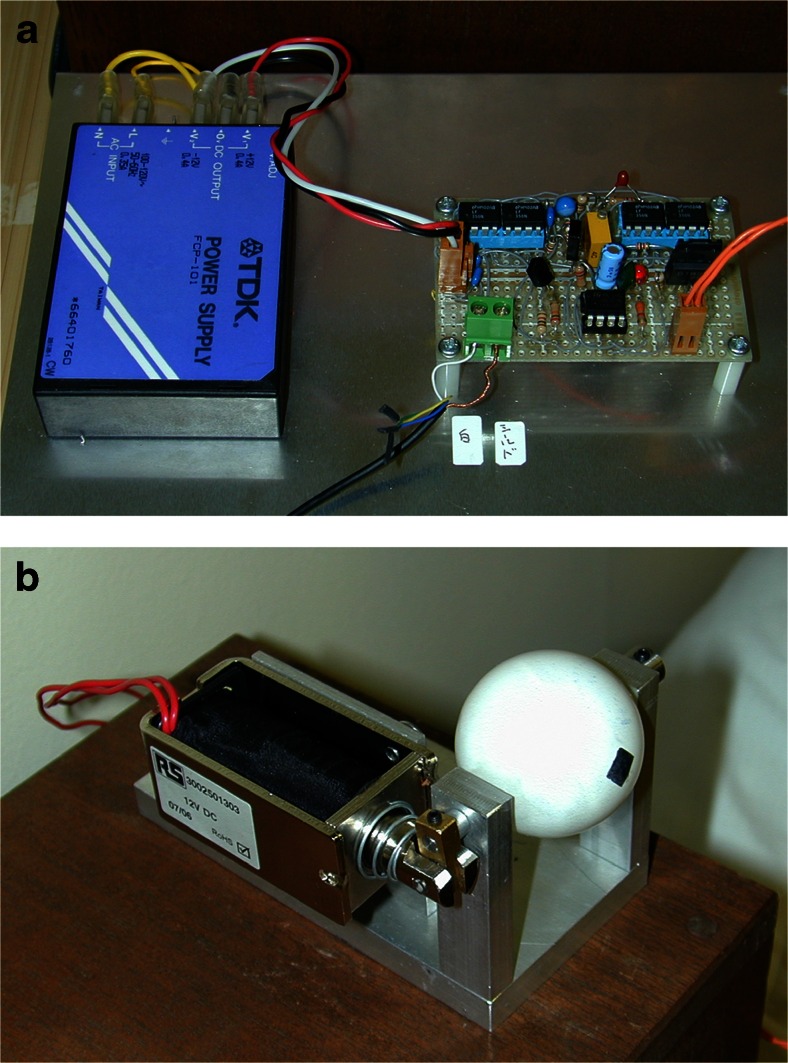
**a**, **b** Circuit and appearance of the blinking periorbital prosthesis model

We tested the operation of our blinking model, which did not precisely reproduce the shape of the eyelid. Surface EMG signals obtained from each subject were filtered with a frequency filter to reduce noise and to increase detectability of signals. The circuit was designed to send a 12-V rectangular pulse to the solenoid when an EMG spike exceeded the predetermined spike detection threshold. The threshold was carefully adjusted for each measurement so that detection omission and noise detection could be minimized (Fig. 2a, b).

## Results

### EMG measurement of the orbicularis oculi muscle and selection of the recording electrode position

**Fig. 3:**
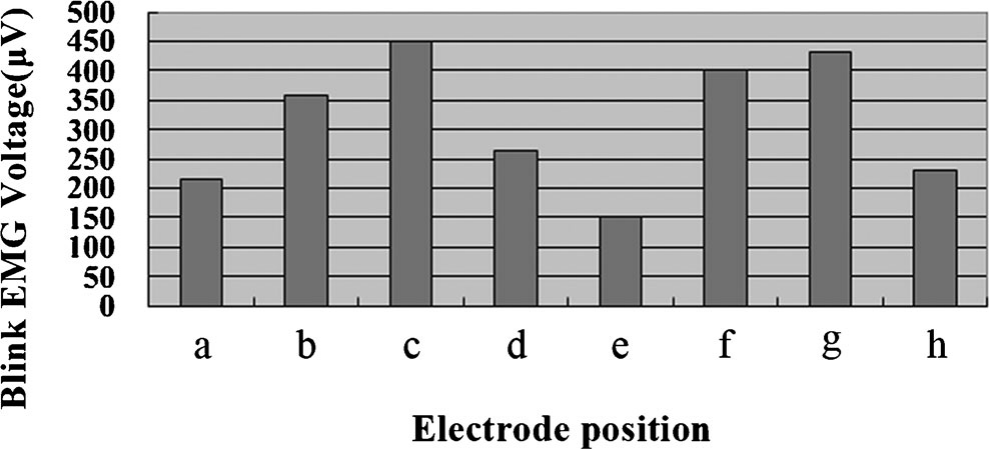
Surface EMG signals of the orbicularis oculi muscle while blinking were measurable at all eight recording electrode positions. However, blink EMG voltages varied with the electrode positions. The mean voltage was 313.3 μV for the eight electrode positions

Surface EMG signals of the orbicularis oculi muscle while blinking were measurable at all eight recording electrode positions. The mean blink EMG voltage was 313.3 μV for the eight positions (Table 1) (Fig. 3). However, the voltages varied with the electrode positions. The mean voltage was highest at the central lower eyelid (449.9 μV), followed by the central upper eyelid (430.8 μV) and lowest at the medial canthus (152.9 μV).

**Table 1:** Surface measurement of blink EMG voltage of the orbicularis oculi muscle at eight recording electrode positions in four healthy subjects

Electrode position	Blink EMG voltage (μV)	
	Mean	Maximum
a (Lateral canthus)	212.5	300.7
b (Lateral lower eyelid)	358.0	536.6
c (Central lower eyelid)	449.9	949.8
d (Medial lower eyelid)	265.1	407.0
e (Medial canthus)	152.9	363.2
f (Medial upper eyelid)	401.9	668.8
g (Central upper eyelid)	430.8	976.5
h (Lateral upper eyelid)	231.3	432.6

The mean voltage was 313.3 μV for the eight electrode positions

*EMG* electromyography

The medial canthus was selected as the recording electrode position for the subsequent EMG measurements on the basis of the abovementioned EMG data and with consideration for the esthetic appearance of a prosthesis. If the prosthesis is designed to use EMG signals through surface electrodes attached to the intact eyelid, the electrodes should not be conspicuous. To give a natural appearance to the blinking prosthesis, we plan to create an eyeglass-type prosthesis that conceals lead wires within the eyeglass frame and has electrodes embedded in a nose pad at the medial canthus.

### Assessment of EMG cross talk

**Fig. 4:**
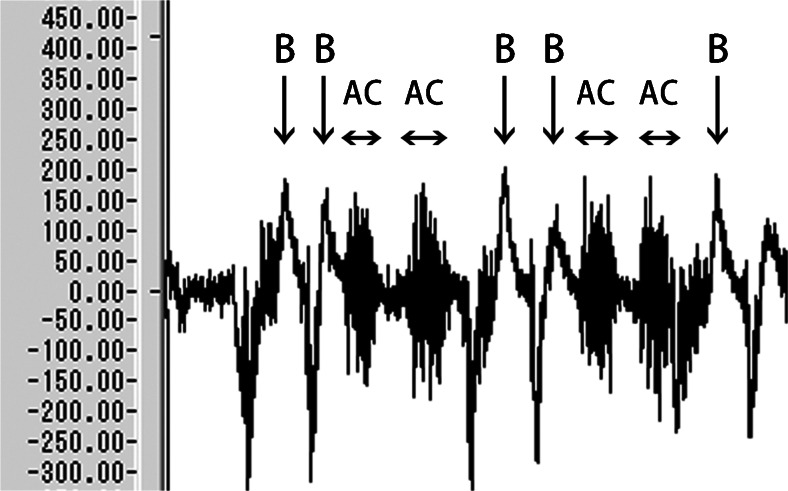
Blink EMG voltage (*B*) and artifact voltage induced by clenching (*Ac*). The mean voltage of artifact induced by clenching was 96.5 μV, which was lower than mean blink EMG voltage (152.9 μV). However, the maximum artifact voltage (175.5 μV) exceeded mean blink EMG

The mean artifact voltage induced by clenching was 96.5 μV (Fig. 4), which was lower than the mean blink EMG voltage measured at the medial canthus (152.9 μV). However, the maximum artifact voltage (188.5 μV) exceeded the lowest mean blink EMG.

**Fig. 5:**
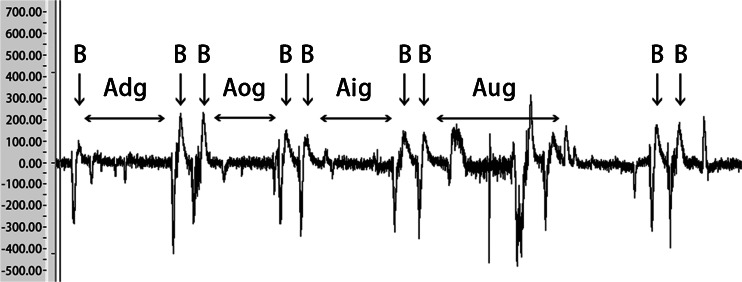
Blink EMG voltage (*B*) and artifact voltages induced by ocular movements, *Aig* artifact induced by inward gaze, *Aog* artifact induced by outward gaze, *Adg* artifact induced by downward gaze, *Aug* artifact induced by upward gaze

Measurement of artifact induced by ocular movements (Fig. 5) showed no major differences between downward, inward, and outward gazes. For these ocular movements, the mean artifact voltage was 25.5 μV and the maximum artifact voltage was 65.7 μV. The artifacts were easily distinguishable from blink EMG signals. However, for upward gaze, artifacts occurred from the frontalis muscles when the subject lifted his or her eyebrows. The maximum artifact voltage was as high as 189.5 μV. These results suggest that artifacts induced by upward gaze could affect the performance of our prosthesis model under the present experimental conditions.

**Fig. 6:**
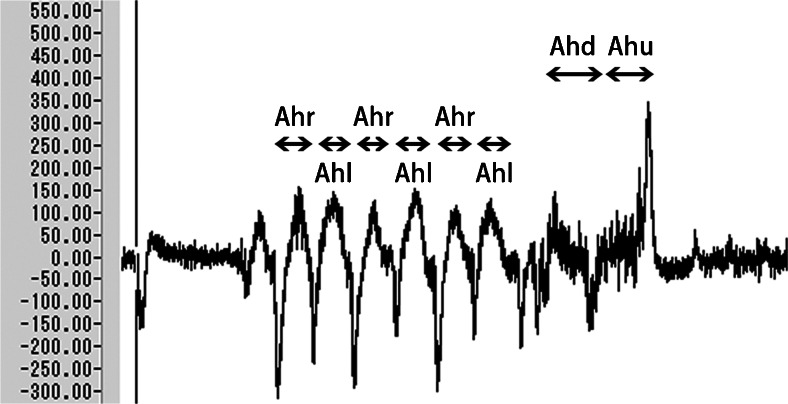
Artifact induced by headshakes. *Ahr* Artifact induced by turning the head right, *Ahl* artifact induced by turning the head left, *Ahd* artifact induced by turning the head down, *Ahu* artifact induced by turning the head up

The maximum artifact voltage was 151.5 μV for a horizontal headshake and 348.8 μV for a vertical headshake (Fig. 6). This result suggests that artifacts induced by a headshake could affect the performance of our prosthesis model under the present experimental conditions. The maximum voltage of artifact induced by speaking was 95.8 μV, which was negligible.

### Operation verification of the prosthesis model

**Fig. 7:**
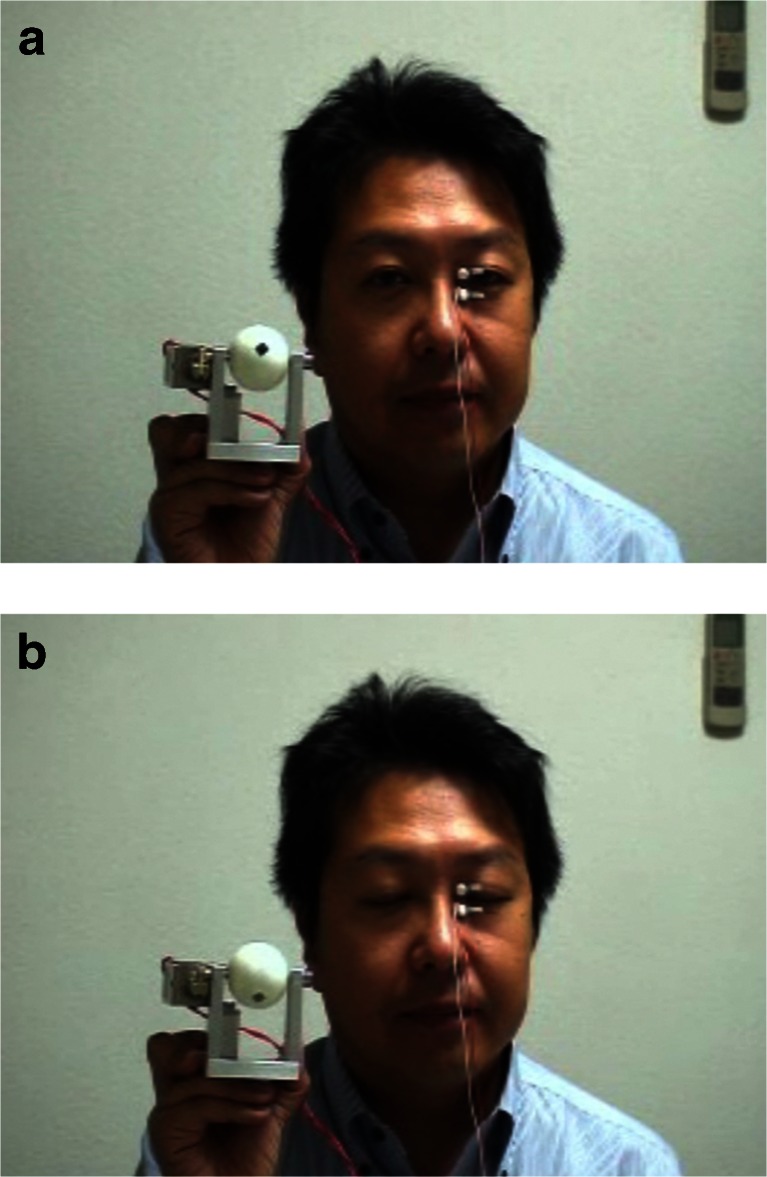
**a**, **b** and Video 1 Video of operation verification of the blinking periorbital prosthesis model. Under the conditions without cross talk, the prosthesis model detected and synchronized with almost all blinks of the intact eyelid. However, the blink synchronization was slightly delayed. Under the conditions with cross talk, the prosthesis model was likely to malfunction. A vertical headshake generated artifacts and caused malfunction of the model, whereas jaw opening–closing movements did not cause malfunction unless the subject clenched as tightly as possible

Based on these results, we tested the operation of the blinking model and found it successful. When artifacts were excluded as much as possible, the model detected and synchronized with almost all blinks of the intact eyelid. However, the blink synchronization was slightly delayed (Video 1) (Fig. 7a, b).

Under the conditions that artifacts were generated, some artifacts caused faulty operation of the model. A vertical headshake generated artifacts and frequently caused malfunctions. Jaw opening–closing movements did not cause malfunction unless the subject clenched as tightly as possible.

## Discussion

A single blink normally takes 200 to 300 ms, and eye opening is usually longer than eye closing (approximately 80 ms) [13]. On average, blink frequency is 5 to 20 times per minute but varies widely due to factors such as conjunctival stimulation caused by wind as well as the level of concentration [14].

Recent prosthetic limbs use direct control or pattern recognition control for myoelectric limb control [15]. Direct control—the conventional method—uses differences in EMG amplitudes between a pair of agonist–antagonist muscles to control movements of a single degree of freedom (DOF). Direct control needs no EMG waveform analysis because the method uses EMG amplitudes to detect the patient’s movement intention. This advantage allows for a simply structured myoelectric prosthesis and prevents delays caused by EMG waveform analysis [3, 12].

On the other hand, the newer method of pattern recognition control detects a patient’s movement intention by analyzing the wave patterns of EMG signals extracted from the target muscle. A computer analyzes the EMG patterns and commands the prosthesis to perform a movement according to predetermined algorithms [12, 1]. This method allows the prosthesis to move at more degrees of freedom than the number of EMG electrode placements [2]. However, waveform analysis and command output may delay movements of the prosthesis.

In this study, our blinking model used direct control. The eyelid movement from start to end of each blink was always assumed to be the same. Therefore, the device was designed to detect the trigger signal only for starting the blink, and each blink of the prosthesis was assumed to be completed automatically. This enabled us to design the system requiring EMG measurement at only a single site, the orbicularis oculi muscle.

If the system were designed to accurately reproduce a 1 DOF motion of eyelid movement, natural movements other than blinking could be added, such as keeping the eyelid closed halfway or completely. However, to follow the DC strategy faithfully, EMG signals should also be obtained from the antagonist levator palpebrae muscle. To our knowledge, previous reports on electrode implantation into the eyelid are limited to the orbicularis oculi muscle in rabbits [16] and dogs [17, 18].

In the present study, our model had difficulty in separating EMG signals of the orbicularis oculi muscle from artifacts from the masticatory muscles due to tight clenching, artifacts from the frontalis muscles, and artifacts due to a vertical headshake. Such EMG cross talk can be avoided by placing the recording electrodes on the central lower eyelid, where the mean blink EMG voltage (449.9 μV) was highest of the eight electrode positions tested. If the central lower or upper eyelid is selected for the electrode position, small, thin, inconspicuous electrodes and lead wires should be developed.

Another prosthetic challenge is delay of blinking of the artificial eyelid. In the present study, our blinking model was synchronized with the blink of the subject’s eyelid, but the model lagged slightly behind. One of the possible causes of the delay is that the model used a peak EMG signal as a trigger for blinking. A peak EMG signal indicates that the number of muscle fibers mobilized for muscle movements has reached its peak. This suggests that a peak EMG signal is generated immediately before the completion of eyelid closure. In other words, the signal is generated 50 to 80 ms after blinking begins. The delay could be minimized if a blinking prosthesis used pattern recognition control detecting EMG wave patterns at the initiation of blinking. However, this method may cause another delay due to pattern analysis with a computer.

In 1999, Klein et al. reported a study that was similar to our present study, but they used needle electrodes to detect EMG signals from the orbicularis oculi muscle [9]. Needle EMG is less likely to be interfered with artifacts than surface EMG because needle EMG signals are larger than surface EMG signals. However, for practical use, we believe that blinking prostheses should use surface EMG rather than needle EMG.

Honda et al. reported that they treated a patient with unilateral orbital resection successfully with their blinking orbital prosthesis using surface EMG signals of the residual orbicularis oculi muscle on the affected side [8]. The report did not mention time lag in synchronization or cross talk between the target muscle and other muscles. The researchers described that surface EMG signals of the residual orbicularis oculi muscle on the affected side were smaller than those on the contralateral side. On the basis of this finding and our study results, we believe that Honda’s prosthesis could not avoid artifacts. However, using the signal from the affected side could probably conceal surface electrodes behind the prosthesis.

## Conclusion

We developed a blinking prosthesis model that synchronizes with blinking of the intact eyelid by using surface EMG signals of the orbicularis oculi muscle on the nonaffected side. The amplitude of surface EMG signals of the target muscle was sufficiently high for the application of blinking prostheses in practical use. Our prosthesis model successfully synchronized with blinks of the intact eyelid under experimental conditions without cross talk between the orbicularis oculi muscle and other facial muscles. If cross talk can be minimized, the use of surface EMG signals would be the most promising approach to applying blinking facial prostheses in practical use.

## Electronic supplementary material

ESM 1 (MPG 3122 kb)
